# Erchen Decoction and Linguizhugan Decoction Ameliorate Hepatic Insulin Resistance by Inhibiting IRS-1Ser307 Phosphorylation In Vivo and In Vitro

**DOI:** 10.1155/2017/1589871

**Published:** 2017-05-29

**Authors:** Huicun Zhang, Na Ta, Pengmin Chen, Hongbing Wang

**Affiliations:** ^1^Beijing Hospital of Traditional Chinese Medicine, Capital Medical University, Beijing 100010, China; ^2^Beijing Institute of Traditional Chinese Medicine, Beijing 100010, China; ^3^China National Health Development Research Center, Beijing 100019, China; ^4^China-Japan Friendship Hospital, Beijing 100029, China

## Abstract

Erchen decoction (ECD) and Linguizhugan decoction (LGZGD), both are Chinese herbal formula, have been used clinically for the treatment of nonalcoholic fatty liver disease (NAFLD). However, their therapeutic mechanisms are still unclear. Because insulin resistance (IR) is a key etiological factor in the pathology of high-fat diet- (HFD-) induced NAFLD, in this study, the protective effects of ECD and LGZGD on HFD-induced insulin resistance in rats were evaluated and their mechanisms were investigated by OGTT and Western blot. The results showed that treatment with ECD and LGZGD significantly improved insulin resistance and liver damage in rats, evidenced by supported serum aminotransferase levels and the histopathological examination. ECD and LGZGD also showed significant protective effects against HFD-induced hyperlipidemia and the inhibition of the hepatocyte proliferation by palmitate. Furthermore, supplementation of ECD and LGZGD decreased TNF-*α*, NF-*κ*B, and IRS-1Ser307 phosphorylation expressions in vivo and in vitro. These results indicated that ECD and LGZGD have protective effects against HFD-induced liver IR and their underlying mechanisms involve the TNF-*α* and insulin pathway. These findings would be beneficial for understanding of the therapeutic effects of ECD and LGZGD in treatment of NAFLD.

## 1. Introduction

Nonalcoholic fatty liver disease (NAFLD) is a disease located in hepatic lobule, and its pathological characteristics are the liver cells diffuse fatty degeneration and fat accumulation without excessive drinking history of clinical syndrome. NAFLD is composed of four pathological processes including simple fatty liver, fatty liver, fatty liver fibrosis, and fatty liver cirrhosis. At present, the disease may belong to a genetic environment-metabolic stress related diseases [1–4].

NAFLD is often associated with overweight (obesity), type 2 diabetes, and hyperlipidemia and other metabolic disorders which are related to insulin resistance (IR). The “two strikes” theory considers that insulin resistance is the first hit in the development of NAFLD and degeneration of hepatocytes is very sensitive to damage factors; the second attack comes from oxidative stress and lipid peroxidation and inflammation, which result in the second hit. At present, the research concerning NAFLD pathogenesis is mainly focused on IR, because IR is the potential abnormal factor in most patients. IR is initiating and the central part in the pathogenesis of NAFLD [5–8].

The theory of traditional Chinese medicine believes that NAFLD is due to eating too much fat and generates the disorder of spleen and stomach's transportation. Liver fails to disperse phlegm turbidity and phlegm turbidity is formed. Phlegm turbidity is obstructed in the liver and formed NAFLD at last. ECD's function is removing dampness and phlegm. ECD is partial to dryness; LGZGD's function is warming drink and strengthening spleen to eliminate dampness. LGZGD is partial to warm. They are both widely used in treating phlegm production of spleen. ECD and LGZGD are widely applied for the treatment of the digestive, cardiovascular, and respiratory system diseases. At present, there are few researches concerning ECD and LGZGD in the treatment of insulin resistance of NAFLD, so we intend to study the similarities and differences of molecular mechanisms of ECD and LGZGD in treatment of insulin resistance of NAFLD; and it may provide theoretical guidance to clinical application for both decoctions.

## 2. Materials and Methods

### 2.1. Laboratory Animals and Cells

Experimental SD rats (Beijing Weitong Lihua Research Center for Experimental Animals)and hepatocytes of rats (BRL) were purchased from cell resource center of Shanghai Institutes for Biological Sciences, Chinese Academy of Sciences.

### 2.2. Main Reagents and Drugs

DMEM medium and Fetal calf serum were obtained from GE Healthcare; anti-TNF-*α* antibody (sc-1350) and anti-NF-*κ*B antibody (sc-372) were obtained from Santa Cruz Biotechnology; anti-IRS-1Ser307 antibody (AI623) was obtained from Beyotime Biotechnology, China, and anti-GAPDH (TA-08) and ECL display color liquid were purchased from Beijing Zhongshan Jinqiao Biological Technology Co., Ltd., China.

### 2.3. Preparation of ECD and LGZGD

ECD consists of four dried crude herbs listed as follows:* Pinellia ternata*, Pericarpium Citri Reticulatae,* Poria cocos*, and licorice. The ratio of herbs* Pinellia ternata*, Pericarpium Citri Reticulatae,* Poria cocos*, and licorice is 5 : 5 : 3 : 2. LGZGD is composed of* Poria cocos*, cassia twig, Rhizoma Atractylodis Macrocephalae, and licorice, and their ratio is 4 : 3 : 2 : 2. On the basis of standards specified in the Chinese Pharmacopoeia (2010 edition), all the herbs were provided by Beijing Tong Ren Tang Medicinal Materials company. ECD and LGZGD were prepared in our laboratory from a mixture of chopped crude herbs, extracted in distilled water at 100°C for 2 h. ECD solution was condensed to the density of 0.9 g/crude herb/ml, while the extract LGZGD had liquid density of 0.66 g/crude herb/ml and was stored at −20°C until further use.

### 2.4. In Vivo Experimental Design

Forty male Sprague-Dawley (SD) rats, weighing 120 ± 20 g, were obtained from Beijing Weitong Lihua Research Center for Experimental Animals; they were maintained in a temperature-controlled room (25 ± 1°C on a 12 h : 12 h light-dark cycle) in the animal center (Beijing Hospital of Traditional Chinese Medicine, Capital Medical University, Beijing, China). The study was carried out under the guidelines for animal experimentation set by them, and the protocol was approved by the animal studies ethics committee of Beijing Hospital of Traditional Chinese Medicine, Capital Medical University.

After acclimation for a week, forty rats were randomly divided into five groups of 8 rats each. One group (normal diet, ND, *n* = 8) of rats were fed with 11.4% kcal fat diet (Beijing science and cooperation Feed Technology Limited Company, Beijing, China; protein: 27.5%, carbohydrate: 65.8%, and fat: 11.4% kcal/g), and the other four groups (high-fat diet, HFD, *n* = 8; high-fat diet along with ROG and HFD + ROG, *n* = 8; high-fat diet along with ECD and HFD + ECD, *n* = 8; and high-fat diet along with LGZGD and HFD + LGZGD, *n* = 8) were fed with 33.1% kcal fat diet (Beijing science and cooperation Feed Technology Limited Company, Beijing, China; protein: 19.6%, carbohydrate: 47.1%, and fat: 33.1% kcal/g). High-fat diet lasted for 8 weeks to establish NAFLD rat model [9, 10]. From the ninth week, rats were dosed by oral gavage once per day for 4 weeks with ROG, ECD, and LGZGD of 5 ml/kg·d. After 4 weeks of treatment, blood samples were taken from the rats after anesthesia. The rats were then sacrificed and the liver was removed and stored at −80°C for subsequent analysis.

### 2.5. Serum Biochemical Parameter Analysis

The analysis of serum including alanine aminotransferase (ALT) and aspartate aminotransferase (AST) and levels of blood glucose (GLU), triglyceride (TG), total cholesterol (TC), low-density lipoprotein cholesterol (LDL), and high-density lipoprotein cholesterol (HDL) were measured by 7160 automatic biochemical analyzer (Hitachi, Japan) according to the manufacturer's instructions.

### 2.6. Histological Examination and Assessment

Sections of the liver samples (4 *μ*m thick) or the frozen liver tissues (5 *μ*m thick) were stained with hematoxylin-eosin (H&E) or Oil Red O and were examined under light microscope (Olympus Medical Systems Corp., Tokyo, Japan). Nonalcoholic fatty liver disease activity score was used to evaluate seriousness of NAFLD. Steatosis (on a scale from 0 to 3), lobular inflammation (on a scale from 0 to 3), and hepatocellular ballooning (on a scale from 0 to 2) are the foundation of assessment system. Higher score demonstrates increasing severity of the disease [11].

### 2.7. Liver Index Detection

Liver index was calculated as a ratio (%) of the organ weight (g) to body weight (g) [12].

### 2.8. Oral Glucose Tolerance Test (OGTT)

At the 12th weekend, rats were fasted for 6 hours and 2 g/kg of glucose was orally administered. Then, instant blood sugar apparatus was used to measure blood samples collected from tail veins at 0 min (without glucose load), 60 min, and 120 min (after glucose load) [13].

### 2.9. Insulin Resistance Index

Insulin resistance was calculated by means of the homeostatic model assessment index (HOMAIR). HOMA-IR = [fasting blood glucose (FBG) *∗* fasting insulin (FINS)]/22.5 [14].

### 2.10. Preparation of ROG-, ECD-, and LGZGD-Containing Serum

ROG-containing serum, ECD-containing serum, and Linguizhugan-containing serum groups rats were given ROG, ECD, and LGZGD by oral gavage individually twice a day for 3 consecutive days (0.5 ml/100 g body weight/time); blood was collected 1 h after the last administration via abdominal aorta and then centrifuged. Serum of the same group was pooled, filtered through 0.22 um filter, inactivated at 56°C for 30 minutes, split, and stored at −80°C [15].

### 2.11. Cell Culturing

BRL cells were cultured in DMEM containing 10% fetal bovine serum with 5% CO_2_ in a cell culture incubator at 37°C.

### 2.12. Palmitate on the Proliferation of BRL Cells

BRL cells were plated into 96-well plate at an initial concentration of 5000 cells/hole. When BRL cell reached 60% confluence, BRL cells were followed by the addition of different final concentrations of palmitate (PA) (0, 0.05, 0.1, 0.2, 0.25, and 0.5 mM) culture medium. BRL cells that were incubated with normal culture medium for 24 h and 48 h were used as negative controls in the in vitro experiments. After 24 hours and 48 hours of coculture with PA, the BRL cells' proliferation was measured by MTT assay.

### 2.13. ROG-, ECD-, and LGZGD-Containing Serum on the BRL Cells Viability Assay

BRL cells were plated into 96-well plate at an initial concentration of 5000 cells/hole. When BRL cell reached 60% confluence, BRL cells were divided into 5 groups: ND group, HFD group, ROG group, ECD group, and LGZGD group: the ND group, DMEM with 20% rat serum; HFD group, containing 20% rat serum and 0.25 mM palmitate; ROG group, 20% ROG containing serum and 0.25 mM palmitate; ECD group, 20% ECD containing serum and 0.25 mM palmitate; LGZGD group, 20% LGZGD containing serum and 0.25 mM palmitate. After 24 hours and 48 hours of the coculture, BRL cells' proliferation was measured by MTT assay. The OD values were compared to determine whether the ECD- and LGZGD-containing serums have an effect on cell viability.

### 2.14. In Vitro Experimental Design

After culturing for 24–48 hours in 6-well plates, BRL cells were divided into 5 groups: ND group, HFD group, ROG group, ECD group, and LGZGD group (just as mentioned above). Each group of cells was incubated at 37°C with 5% CO_2_ for 48 hours and then total protein was extracted.

### 2.15. Western Blot Analysis

#### 2.15.1. Protein Extract

The liver tissue and BRL cells treated as above were collected and lysed in ice-cold RIPA lysis buffer (Beyotime, Shanghai, China) for 30 minutes. Protein extracted by lysis buffer was transferred into a precooled EP tube and centrifuged at 20000 r/min × 10 min at 4°C. The supernatant was immediately transferred into a precooled EP tube and mixed with an equal volume of buffer solution. The sample was subsequently boiled at 100°C for 10 min and stored at −20°C. A portion was used to determine the lysate protein concentration via the BCA (Beyotime Biotechnology, China) method, and another portion was stored at −80°C.

#### 2.15.2. Protein Analysis

80 *μ*g liver and 30 *μ*g BRL cells denatured protein liquid were separated by 10% SDS gel electrophoresis (SDS-PAGE). The proteins from the gel were then transferred to nitrocellulose membrane. The membrane was blocked with 8% nonfat milk in TBST at room temperature for 1 h and then anti-TNF-*α* antibody (sc-1350), anti-NF-*κ*B antibody (sc-372), anti-IRS-1Ser307 phosphorylation antibody (AI623), and anti-GAPDH antibody (TA-08) were added and incubated at 4°C overnight. After the membrane was washed three times for 10 min each in TBST, secondary antibody was added, and the membrane was incubated at 37°C for 1 h. The membrane was washed again, stained with ECL, and exposed to X-ray film. Image analysis was then performed.

### 2.16. Statistical Analysis

ImageJ analysis software was used to conduct the image analysis. Each image analysis result was represented as means ± standard deviation ($\overset{-}{x}\pm s$), and a one-way analysis of variance was used to analyze data in the statistical software package SPSS 17.0. The results were considered significantly different when *P* < 0.05.

## 3. Results

### 3.1. Effects of ECD and LGZGD on Body Weight and Liver Index of HFD-Fed Rats

**Figure 1:**
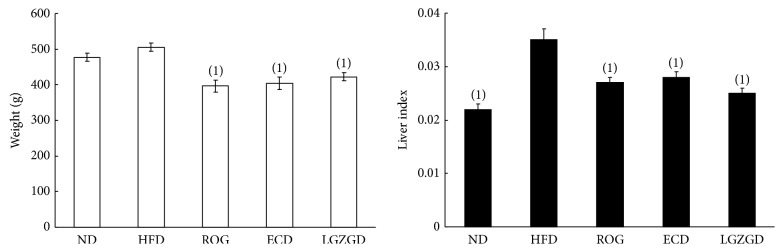
Rat's body weight and liver index. Compared with the HFD group, ^(1)^*P* < 0.05.

At the end of the 12th week, there was a significant difference in the body weight between the treatment and HFD groups (Figure 1). Compared with the ND group, the liver index of the rats in the HFD group was elevated (*P* < 0.05). ROG, ECD, and LGZGD significantly decreased liver index, and no significant difference existed in the liver index of the rats in the treatment group (Figure 1).

### 3.2. Effects of ECD and LGZGD on Liver Morphology and Histopathology

**Figure 2:**
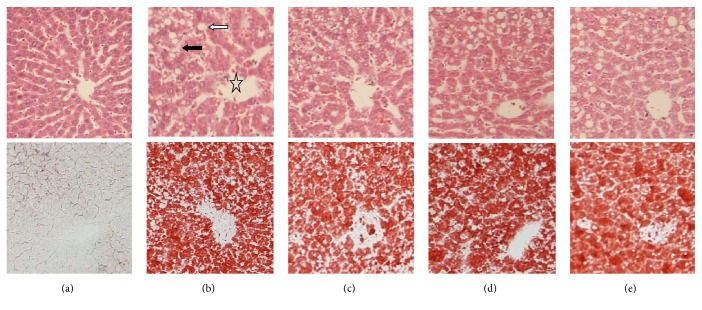
Effect of ECD and LGZGD on hepatic morphology and pathological changes. Histological micrograph of liver specimen from ND, HFD, HFD + ROG, HFD + ECD, and HFD + LGZGD rats (H&E staining and Oil Red O staining, magnification ×100). (a) ND group; (b) the HFD group; (c) the HFD + ROG group; (d) the HFD + ECD group; (e) the HFD + LGZGD group. The major histopathological change induced by HFD in rat's liver was hepatocyte steatosis (filled arrow) with inflammation (open pentagram) and ballooning (white arrow).

H&E and oil red O stained sections were used to obverse the livers' histological changes. HFD group rats' liver tissue showed abundant fat deposition and mononuclear inflammatory cells infiltration, and a large number of fat vacuoles and ballooning existed in cytoplasm (Figure 2). Histological examination showed that fatty degeneration, inflammation, and hepatocyte ballooning in ECD group, LGZGD group, and ROG group were alleviated compared to HFD group (Table 1).

### 3.3. Effects of ECD and LGZGD on Serum Lipids, ALT, and AST Activities of HFD-Fed Rats

At the end of administration, the serum TC, TG, LDL, ALT and AST levels of the rats in the HFD group were significantly increased compared to those of ND group (*P* < 0.05), while serum HDL level was reduced in the HFD group rats (*P* < 0.05). ROG, ECD, and LGZGD lowered TG, TC, and LDL levels when compared with the HFD group (*P* < 0.05). ROG, ECD, and LGZGD failed to effectively increase HDL (*P* > 0.05). Compared with the HFD group, except ECD, ROG and LGZGD significantly decreased the serum ALT and AST levels (*P* < 0.05), and there was no significant difference between ROG and LGZGD groups. ECD significantly decreased the serum ALT without AST (*P* < 0.05), suggesting that ECD and LGZGD could protect the liver injury induced by the HFD feeding (Table 2).

### 3.4. Effect of ECD and LGZGD on Oral Glucose Tolerance Test in HFD-Fed Rats

After overnight fasting for 12 h, there existed no difference in blood glucose of each group. One hour after intragastric administration of glucose, ROG group, ECD group, and LGZGD group's blood glucose was significantly reduced compared to HFD group (*P* < 0.05), and there was no significant difference among the three groups (*P* > 0.05); after 120 min, HFD group's blood glucose was still significantly higher than other groups (*P* < 0.05) and blood glucose of ECD group was significantly higher than LGZGD group (*P* < 0.05).

### 3.5. Effect of ECD and LGZGD on Insulin Resistance Index in Rats with Nonalcoholic Fatty Liver Disease

Compared with the ND group, insulin resistance index of HFD group was obviously higher (*P* < 0.05). Compared with HFD group, insulin resistance index of ROG, ECD, and LGZGD groups was decreased (*P* < 0.05) (Figure 4).

### 3.6. The Inhibition of Palmitate on Proliferation of Hepatocytes

After being incubated with palmitate for 24 h, the cells proliferation was decreased significantly starting from 0.2 mM palmitate. Meanwhile after 48 hours of being stimulated with palmitate, 0.1–0.5 mM palmitate significantly inhibited the liver cells proliferation (Table 3). The results showed inhibition of palmitate on liver cell proliferation with time- and dose-dependence.

### 3.7. ECD- and LGZGD-Containing Serum Resists Inhibition of Palmitate on Hepatocytes Viability

After being stimulated with palmitate and incubated with it for 24 h, the cells proliferation was decreased significantly. ECD and LGZGD groups had significant (*P* < 0.05) effect on the cellular proliferation when compared with HFD group. That means ECD and LGZGD can significantly antagonize 0.25 mM palmitate's inhibition on proliferation of BRL. While being stimulated with palmitate lasted 48 hours, only ROG- and LGZGD-containing serum can effectively antagonize inhibition of palmitate when compared with HFD group (Table 4).

### 3.8. Effects of ECD and LGZGD on TNF-*α*, NF-*κ*B, and IRS-1Ser307 Phosphorylation Expressions in Hepatic Tissues of HFD-Fed Rats

Hepatic total TNF-*α*, NF-*κ*B, and IRS-1Ser307 phosphorylation expressions of HFD group's rats were significantly higher than those in ND group's rats (*P* < 0.05), but they were decreased in rats in ROG, ECD, and LGZGD group. NF-*κ*B and IRS-1Ser307 phosphorylation expressions of rats in ROG group were more than those of rats in ECD and LGZGD groups and there was no difference between rats of ECD group and rats of LGZGD group (*P* > 0.05, Figure 5).

### 3.9. Effects of ECD- and LGZGD-Containing Serum on TNF-*α*, NF-*κ*B, and IRS-1Ser307 Phosphorylation Expressions in BRL Cells Stimulated with Palmitate

As shown in Figure 7, in BRL cells being stimulated with palmitate for 48 h, TNF-*α*, NF-*κ*B, and IRS-1Ser307 phosphorylations in HFD group were overexpressed; ROG-, ECD-, and LGZGD-containing serum could decrease TNF-*α*, NF-*κ*B, and IRS-1Ser307 phosphorylation expressions. Except IRS-1Ser307 phosphorylation, there was significant difference (*P* < 0.05) in TNF-*α* and NF-*κ*B expressions between ECD- and LGZGD-containing serums (Figure 6).

## 4. Discussion

Our present study showed that the administration of ECD and LGZGD has preventive effect against hepatic insulin resistance in vivo and in vitro by decreasing of IRS-1Ser307 and TNF-*α*.

In China, ECD and LGZGD have been used in clinical practice to alleviate NAFLD. Previous studies showed that ECD and LGZGD could ameliorate dyslipidemia and hepatic steatosis [16, 17]. However, the underlying molecular mechanism needs to be further investigated. IRS-1Ser307 phosphorylation protein activity is recognized as a major regulator of insulin resistance. So, in this study, we observed the effect of ECD and LGZGD on IRS-1Ser307 phosphorylation protein expression and the related pathway in insulin resistance in vitro and in vivo. Rosiglitazone improves insulin resistance by reducing the livers' free fatty acid utilization, TNF-*α* release, and IRS-1Ser307 phosphorylation expression [18–22], so we take rosiglitazone as a positive control.

To evaluate the protective effect of ECD and LGZGD on NAFLD IR injury in vivo, we used HFD for 8 weeks to duplicate fatty liver model in rats. In vivo animal experiments showed that ROG, ECD, and LGZGD could decrease abnormal HOMA-IR caused by HFD. At the same time, ROG, ECD, and LGZGD could improve abnormal blood lipid as follows. For the rats in HFD groups, serum TG, LDL, and serum ALT and AST levels were higher compared to normal control group, while for HFD groups, HDL was lower than normal control group. These results indicated that the fatty liver model with triglycerides accumulation in the liver was induced successfully by the high-fat diet. Using these model rats, we demonstrated that ECD and LGZGD can reduce serum TG, TC, and LDL and there was no obvious difference among them. ROG, ECD, and LGZGD failed to significantly increase HDL (Table 2). ROG and LGZGD can significantly decrease the level of serum ALT and AST levels and LGZGD is better than ROG, while ECD remarkably decreases the level of serum ALT (Table 2).

In this study, compared with the ND group, although the weight of the rats in the HFD group did not increase significantly (*P* > 0.05), the liver index was elevated (*P* < 0.05). The difference of liver index in HFD group and ND group was due to the liver weight of rats in HFD group which was heavier than that of ND group's rats. Some reports found that, when compared with the ND group, the weight of the rats in the HFD group increases significantly. But there was no significant difference between the weight of the rats in the HFD group and that of the rats in the ND group in our study. We ascribed it to the difference of composition of high-fat diet between our study and others. In our study, HFD rats were fed with 33.1% kcal fat diet, while rats in HFD group were fed with 45–60% kcal fat diet in previous studies [23–25].

Insulin signal transduction change is the main factor for the fatty liver. Studies have shown that IRS-1Ser307 phosphorylation plays an important role in mediating negative feedback regulation of insulin transduction. It not only interferes in interaction between IRS-1 and insulin receptor but also hinders the role of IRS-1 tyrosine's phosphorylation. IRS-1 tyrosine's phosphorylation is downstream of insulin signaling. ECD and LGZGD have been used in clinical practice to alleviate NAFLD in China. Previous studies showed that ECD and LGZGD could ameliorate dyslipidemia and hepatic steatosis [16, 17]. However, the underlying molecular mechanism needs to be further investigated.

**Figure 3:**
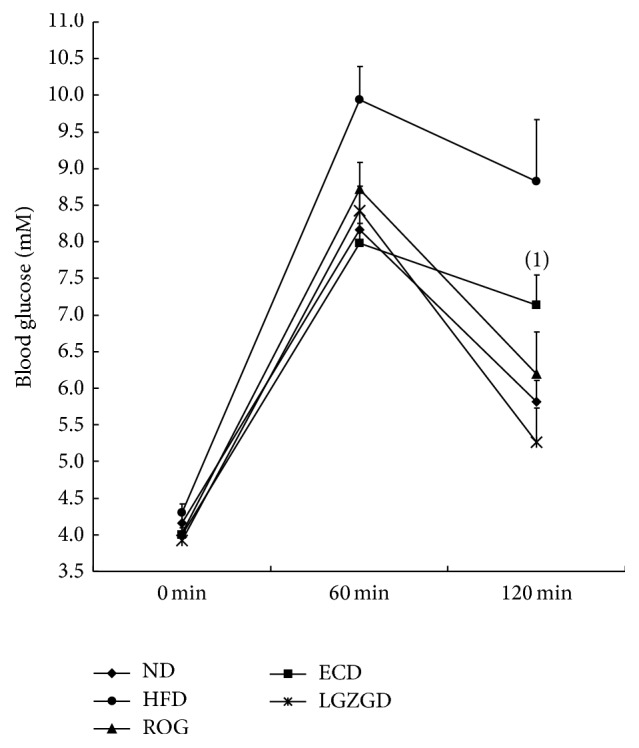
ECD and LGZGD on OGTT. ^(1)^*P* < 0.05 versus the LGZGD group.

**Figure 4:**
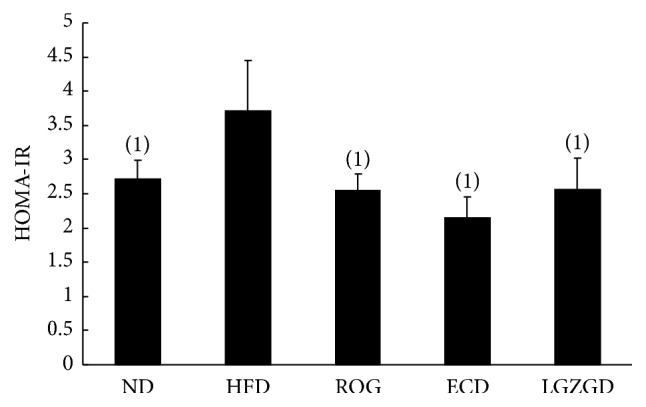
ECD and LGZGD on insulin resistance index. ^(1)^*P* < 0.05 versus the HFD group.

**Figure 5:**
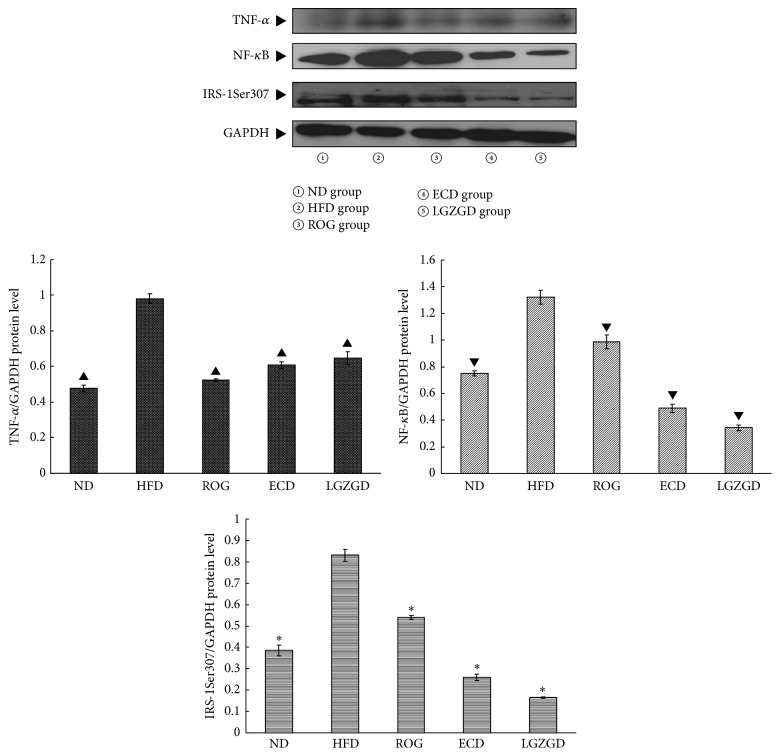
ECD and LGZGD on TNF-*α*, NF-*κ*B, and IRS-1Ser307 phosphorylation expressions in hepatic tissues of HFD-fed rats. ^▲; ▼; **∗**^*P* < 0.05 versus the HFD group.

Considering the key role of IR activation in regulating lipid metabolism, we hypothesized that IR may play a key role in the effects of ECD and LGZGD against hepatic lipid accumulation. So, in this study, we observed the effect of ECD and LGZGD on IR both in vitro and in vivo. This research showed that ECD and LGZGD could decrease insulin resistance index (Figure 5) and improve NAFLD rats' abnormal glucose tolerance caused by high-fat diet. As for oral glucose tolerance test (OGTT), compared with HFD group, ROG, ECD, and LGZGD can effectively reduce 1-hour and 2-hour postprandial blood glucose (*P* < 0.05). 2-Hour postprandial blood sugar of LGZGD group was lower than that of ECD group (Figure 3). These findings indicated that ECD and LGZGD could ameliorate IR induced by HFD.

**Figure 6:**
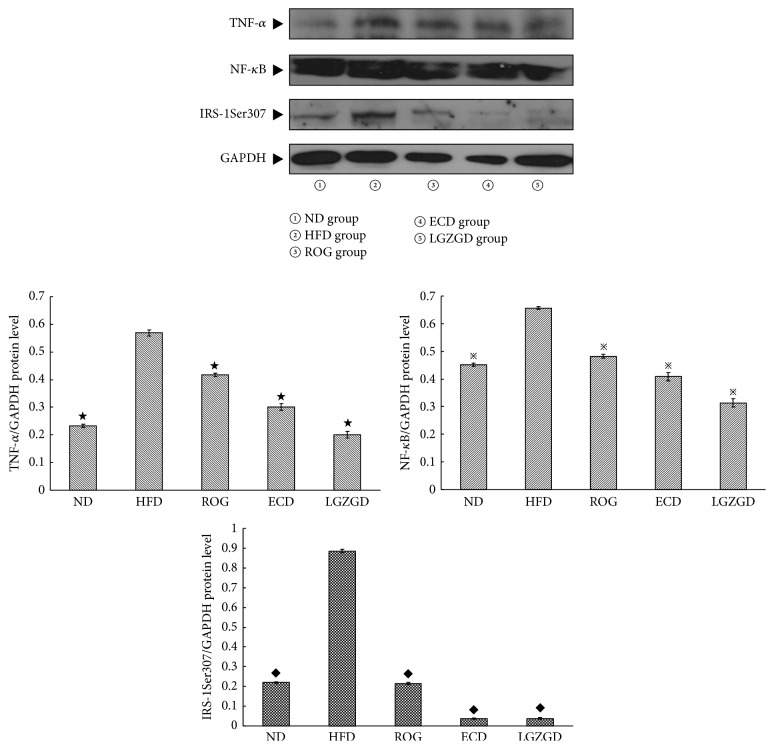
ECD and LGZGD serum containing on TNF-*α*, NF-*κ*B, and IRS-1Ser307 phosphorylation expressions in BRL cells stimulated with palmitate. ^★; **※**; **◆**^*P* < 0.05 versus the HFD group.

Therefore, in order to elucidate some of the underlying mechanisms involved in the protective effects of ECD and LGZGD on HFD-induced IR, the expression of IRS-1Ser307 phosphorylation associated with IR was detected by Western blot. Although ROG can effectively inhibit IRS-1Ser307 phosphorylation caused by HFD, the inhibition ability was weaker than ECD and LGZGD (*P* < 0.05). ECD and LGZGD could significantly inhibit IRS-1Ser307 phosphorylation but inhibition of ECD was weaker than that of LGZGD (Figures 5 and 6).

TNF-*α* and NF-*κ*B are involved in the pathogenesis of NAFLD. TNF-*α* makes IRS-1 serine phosphorylation and reduces the activity of insulin signaling pathway; moreover, TNF-*α* strengthens the lipolysis of adipose tissue and then more free fatty acids promote the secretion of TNF-*α* by TLR4/NF-*κ*B pathway. Excessive TNF-*α* leads to abnormal mitochondria, oxidative stress, fatty acid beta oxidation overload, NF-*κ*B overexpression, and fat deposition in the liver. TNF-*α* and NF-*κ*B interact with each other, and they became vicious circle [26–28]. Therefore, inhibiting NF-*κ*B activation and reducing the expression of TNF-*α* and IRS-1Ser307 phosphorylation were of great significance for the prevention and treatment of NAFLD insulin resistance. ECD and LGZGD seem to reverse the regulation of HFD on these genes.

In order to study further mechanism of ECD and LGZGD improving NAFLD IR in vitro, BRL cells, a kind of rat hepatocytes, were incubated with palmitate. We used 0.25 mM palmitate to stimulate BRL cells for 48 h to establish cellular NAFLD model. Free fatty acids (FFAs) have direct toxic effects on cell function and apoptosis. FFAs are composed of saturated fatty acid and unsaturated fatty acid. Damage of saturated fatty acid is much more than that of unsaturated fatty acids. Palmitate is the main component of saturated fatty acids [29, 30]. Palmitate inhibits on liver cell proliferation with time and dose dependence. The FFA from adipose tissue is the main source of liver fat, which accounts for 62%–82% of TG in liver [31]. Chinese medicines have numerous chemical compositions and possess multiple targets in human body. When traditional Chinese medicines are through stomach, intestine, and liver digestion and absorption, major medicinal effective ingredients go into the blood plasma and the effect of Chinese composite recipe will play a role [15]. Therefore, to reproduce the features of ECD and LGZGD after metabolism in digestive system, we prepared ECD- and LGZGD-containing serum. The same as the results in the animal study, supplementation of ECD and LGZGD decreased the phosphorylation levels of IRS-1Ser307 and TNF-*α* and hepatic nuclear protein expression of NF-*κ*B in BRL cells induced by palmitate.

**Figure 7:**
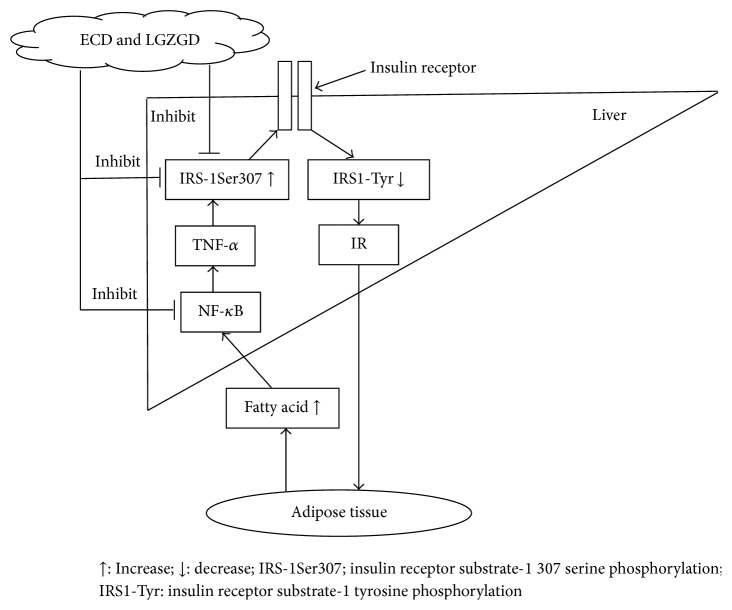
ECD and LGZGD improve hepatic insulin resistance by inhibiting TNF-*α*, NF-*κ*B, and IRS-1Ser307 phosphorylation expressions.

From the preceding discussion, we presume that ECD and LGZGD significantly not only inhibit TNF-*α*, NF-*κ*B, and IRS-1Ser307 phosphorylation expression and improve insulin resistance but also decrease free fatty acids released by adipose tissue lipolysis and then decrease hepatic de novo lipogenesis and fat accumulation (Figure 7).

ECD and LGZGD are prepared from aqueous extracts of 4 medicinal herbs and two of them are the same.* Pinellia ternata*, Pericarpium Citri Reticulatae,* Poria cocos*, licorice, cassia twig, and Rhizoma Atractylodis Macrocephalae have been reported to possess anti-inflammatory effect [32–41]. In addition, cassia twig and Rhizoma Atractylodis Macrocephalae, two main constituents of LGZD, also showed antioxidant, antiadipogenic, and antiobesity activities and modulation of the gut microbial distribution [40]. Therefore, although ECD and LGZGD may contain hundreds of different chemical compounds, their active ingredients responsible for improving IR and liver protective activities are still unclear. Those researches mentioned above may explain ECD and LGZGD's activities partly.

Although ECD and LGZGD did not have any obviously antagonistic effect on the treatment of NAFLD IR in this study, further systematically experimental study is needed to identify whether they have some side effects.

## 5. Conclusion

Based on the above results indicating that the administration of ECD and LGZGD can suppress the development of HFD-induced fatty liver, we presume that inhibition of TNF-*α*, NF-*κ*B, and IRS-1Ser307 phosphorylation expressions might be major contributor to the beneficial effects of ECD and LGZGD on decreasing hepatic lipid accumulation caused by IR. From these, the theoretical basis will be provided for the future clinical drug clinical application.

## Figures and Tables

**Table 1:** Average score of histopathological findings in livers. ($\overset{-}{x}\pm s$, *n* = 5).

Group	Steatosis	Inflammation	Ballooning
ND	0	0	0
HFD	2.00 ± 0.00^a^	2.33 ± 0.52^a^	2.00 ± 0.00^a^
ROG	1.00 ± 0.00^b^	1.17 ± 0.41^b^	1.17 ± 0.75^b^
ECD	1.00 ± 0.72^b^	1.33 ± 0.52^b^	1.16 ± 0.41^b^
LGZGD	1.00 ± 0.56^b^	1.35 ± 0.46^b^	1.18 ± 0.65^b^

Quantitative data are expressed as mean ± SD. Statistical analysis of the data for multiple comparisons was performed by one-way ANOVA.

^a^*P* < 0.05 versus the ND group and ^b^*P* < 0.05 versus the HFD group.

**Table 2:** Rats serum lipid and ALT/AST ($\overset{-}{x}\pm s$, *n* = 8).

Group	TC (mmol/L)	TG (mmol/L)	HDL (mmol/L)	LDL (mmol/L)	ALT (U/L)	AST (U/L)
ND	1.43 ± 0.10^a^	0.58 ± 0.10^a^	1.15 ± 0.085	0.21 ± 0.01^a^	80.2 ± 4.36^a^	175.2 ± 17.82^a^
HFD	2.32 ± 0.47^b^	0.80 ± 0.11^b^	0.78 ± 0.08^b^	0.74 ± 0.19^b^	115.6 ± 16.76^b^	227.0 ± 9.80^b^
ROG	1.27 ± 0.11^a^	0.42 ± 0.01^a^	0.87 ± 0.06^b^	0.29 ± 0.02^a^	98.4 ± 2.27^a^	190.40 ± 8.14^a^
ECD	1.31 ± 0.09^a^	0.40 ± 0.03^a^	0.83 ± 0.05^b^	0.33 ± 0.03^a^	99.8 ± 9.88^a^	207.80 ± 14.14
LGZGD	1.35 ± 0.08^a^	0.34 ± 0.03^a^	0.88 ± 0.09^b^	0.28 ± 0.02^a^	68.2 ± 3.21^a^	142.00 ± 7.416^a^

*Note*. Quantitative data are expressed as mean ± SD. Statistical analysis of the data for multiple comparisons was performed by ANOVA. ^a^*P* < 0.05 versus HFD group; ^b^*P* < 0.05 versus ND group.

**Table 3:** Inhibition of palmitate on hepatocytes' proliferation ($\overset{-}{x}\pm s$, *n* = 6).

Palmitate (mM)	Exposure time of palmitate	
	24 hours	48 hours
0	0.40 ± 0.01	0.51 ± 0.01
0.05	0.40 ± 0.01	0.49 ± 0.01
0.10	0.41 ± 0.01	0.43 ± 0.02^◆^
0.20	0.31 ± 0.01^⋄^	0.41 ± 0.01^◆^
0.25	0.30 ± 0.01^⋄^	0.39 ± 0.01^◆^
0.50	0.17 ± 0.01^⋄^	0.25 ± 0.01^◆^

*Note*. Different concentrations of palmitate group compared with the control group (palmitate concentration is 0 mM): *P* < 0.05. ^⋄,◆^Different concentrations of palmitate group compared with the control group (palmitate concentration is 0 mM): *P* < 0.05.

**Table 4:** ECD and LGZGD's effect on hepatocytes proliferation ($\overset{-}{x}\pm s$, *n* = 6).

Cell grouping	Serum containing effect time	
	24 hours	48 hours
ND group	0.42 ± 0.02^△^	0.53 ± 0.01^▲^
HFD group	0.29 ± 0.01	0.37 ± 0.01
ROG group	0.34 ± 0.01	0.43 ± 0.01^▲^
ECD group	0.36 ± 0.03^△^	0.37 ± 0.02
LGZGD Group	0.35 ± 0.01^△^	0.45 ± 0.02^▲^

*Note*. ^△;▲^ND group and drug-containing serum group compared with the HFD group: *P* < 0.05.

## Abbreviations

IRS-1Ser307 phosphorylation: Insulin receptor substrate-1 Ser307 phosphorylation
TNF-*α*: Tumor necrosis factor-*α*
NF-*κ*B: Nuclear transcription factor-*κ*B
ALT: Alanine aminotransferase
AST: Aspartate aminotransferase
PA: Palmitate
FFA: Free fatty acid
HDL-C: High-density lipoprotein cholesterol
LDL-C: Low-density lipoprotein cholesterol
NAFLD: Nonalcoholic fatty liver disease
NASH: Nonalcoholic steatohepatitis
TC: Total cholesterol
TG: Triglyceride.
